# Military Dietetics

**Published:** 1900-11-10

**Authors:** 


### MILITARY DIETETICS.

The student of military catastrophes, finding that the chief diseases by which armies are decimated are of the zymotic or infectious type, is apt to jump to the conclusion that among the measures necessary for the prevention of epidemics among troops in the field those which aim at controlling the spread of disease-germs are the most important.

There is much reason, however, for believing that the peculiar liability to certain types of infective disease which is so often shown by troops on active service is largely due to increased personal susceptibility, and that this has much to do with the unsuitable diet on which the soldier sometimes has to subsist.

In civil life, where each man "ends" for himself, an automatic balance is soon struck between work, climate, and food. But where the whole thing has to be planned beforehand, and where plans once laid down and carefully bound in red tape have to be adhered to, the matter is very different, and a slight error in the dietary, even in the direction of excess, may lead to much mischief.

Dr. Louis Seaman, late surgeon to the United States Engineers, writing in the New York *Medical Record,* says that a personal experience in two of the latest tropical wars and a study of the statistics of others have led him to the conviction that the most prominent cause in bringing about the development of preventable diseases in both these wars was the misuse of food. Speaking generally, he considers that the majority of the diseases met with among troops serving in the tropics have their origin in improper food, in over feeding, or in the abuse of stimulants, and he suggests a dietary scale containing much less meat and fat than was issued to the United States' troops. He is a strong advocate of dried or smoked meat in place of the fresh, the tinned, and the salted meat so often used, and of the use of sugar as a supplement to the diet when extra work is required, or as a substitute for some of the more bulky carbo-hydrates when for a time economy of transport is essential.

The point of interest, however, is not merely that an improper diet is wasteful, or that it leads to an excess of dyspeptic ailments, but that by so disturbing the digestive tract it leaves the soldier predisposed to the still more serious infective diseases which always dog the steps of armies. There is much probability in this view.

